# SUMO and ubiquitin-dependent XPC exchange drives nucleotide excision repair

**DOI:** 10.1038/ncomms8499

**Published:** 2015-07-07

**Authors:** Loes van Cuijk, Gijsbert J. van Belle, Yasemin Turkyilmaz, Sara L. Poulsen, Roel C. Janssens, Arjan F. Theil, Mariangela Sabatella, Hannes Lans, Niels Mailand, Adriaan B. Houtsmuller, Wim Vermeulen, Jurgen A. Marteijn

**Affiliations:** 1 Department of Genetics, Cancer Genomics Netherlands Erasmus MC, Wytemaweg 80, Rotterdam 3015 CN, The Netherlands; 2 Department of Pathology, Josephine Nefkens Institute, Erasmus MC, Dr Molewaterplein 50, Rotterdam 3015 GE, The Netherlands; 3 Ubiquitin Signaling Group, Protein Signaling Program, The Novo Nordisk Foundation Center for Protein Research, University of Copenhagen, Blegdamsvej 3B, Copenhagen DK-2200, Denmark

## Abstract

XPC recognizes UV-induced DNA lesions and initiates their removal by nucleotide excision repair (NER). Damage recognition in NER is tightly controlled by ubiquitin and SUMO modifications. Recent studies have shown that the SUMO-targeted ubiquitin ligase RNF111 promotes K63-linked ubiquitylation of SUMOylated XPC after DNA damage. However, the exact regulatory function of these modifications *in vivo* remains elusive. Here we show that RNF111 is required for efficient repair of ultraviolet-induced DNA lesions. RNF111-mediated ubiquitylation promotes the release of XPC from damaged DNA after NER initiation, and is needed for stable incorporation of the NER endonucleases XPG and ERCC1/XPF. Our data suggest that RNF111, together with the CRL4^DDB2^ ubiquitin ligase complex, is responsible for sequential XPC ubiquitylation, which regulates the recruitment and release of XPC and is crucial for efficient progression of the NER reaction, thereby providing an extra layer of quality control of NER.

## Abstract (web summary)

The SUMO-targeted ubiquitin ligase RNF111 promotes K63-linked ubiquitylation of SUMOylated XPC after DNA damage. Here the authors show that RNF111 is responsible for sequential XPC ubiquitylation, and RNF111-mediated ubiquitylation promotes the release of XPC from damaged DNA after NER initiation.

## Graphical abstract

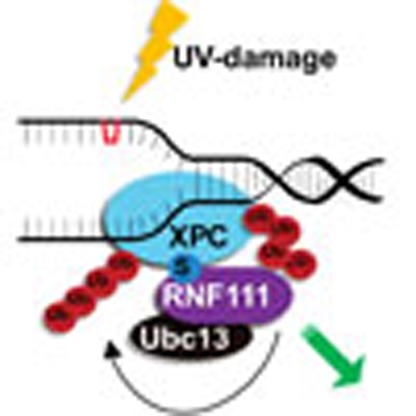

DNA integrity is constantly challenged by internal and external DNA-damaging agents that induce DNA lesions. When not properly repaired, DNA lesions may result in malignant transformation or accelerated ageing. Different DNA repair mechanisms exist that collectively remove most lesions and safeguard genome stability. Nucleotide excision repair (NER) is one of these mechanisms, which removes—in a multistep process—a wide variety of helix-distorting lesions, including ultraviolet (UV)-induced cyclobutane pyrimidine dimers (CPDs) and 6-4 pyrimidine–pyrimidone photoproducts (6-4PPs)^1^. Lesions located in the transcribed strand of active genes block elongating RNA polymerase II and are specifically processed by a dedicated transcription-coupled NER (TC-NER) sub-pathway. However, the vast majority of helix-distorting DNA lesions located anywere in the genome are targeted by the global genome NER sub-pathway (GG-NER)^1^. After damage recognition by one of these sub-pathways, the 10-subunit TFIIH complex is recruited^2^,^3^ to unwind the DNA around the lesion. TFIIH and XPA, which also bind the damaged strand^4^, verify the presence of lesions^5^. Next, RPA binds the undamaged strand and plays a role in correct positioning of the structure-specific endonucleases XPG and ERCC1/XPF to excise a ∼25-nucleotide stretch of single-stranded DNA containing the lesion^6^. The activity of these endonucleases and thereby the excision of the DNA lesion is tightly orchestrated. First, XPG is recruited either independently^7^ or through its interaction with TFIIH^8^. Next, ERCC1/XPF is recruited that can only incise the DNA in the presence of XPG. Only after the 5′ incision has been completed by ERCC1/XPF, the 3′ incision by XPG is triggered^9^. After incision, the DNA is restored to its original state by DNA synthesis and ligation steps.

Within GG-NER, DNA damage recognition occurs through binding of the XPC complex to lesion-induced helix distortions^10^ and is essential for assembly of the core NER factors and progression of the NER reaction^3^. XPC is part of a heterotrimeric complex together with one of the two mammalian orthologs (RAD23A or RAD23B) of the *Saccharomyces cerevisiae* Rad23p^11^ and centrin2 (ref. 12). Although XPC is the main DNA damage sensor of GG-NER, it does not efficiently recognize UV-induced CPDs, which are the most abundant UV-induced DNA lesions. For efficient repair of these lesions initial binding of the UV–DDB complex, a heterodimer consisting of DDB1 and DDB2 (XPE), is required^13^,^14^. UV–DDB is not only involved in damage detection, but together with Cullin-4A (CUL4A) and Rbx1/Roc1 (ref. 15) this complex possesses E3 ubiquitin ligase activity that—amongst others—mediates polyubiquitylation of the DNA damage sensors DDB2 and XPC. As ubiquitin can use all seven internal lysine residues (K6, K11, K27, K29, K33, K48 and K63) and its N terminus for chain formation, different chain linkages can be formed. These various polyubiquitin chain types have distinct structures and different consequences for the target protein^16^. While ubiquitylated DDB2 is targeted for degradation^14^, ubiquitylated XPC is not, but acquires increased affinity for damaged DNA *in vitro*^17^. Following UV irradiation XPC is not only modified by ubiquitin, but also by the small ubiquitin-like modifier (SUMO)^18^,^19^,^20^ in a DDB2- and XPA-dependent manner^19^,^20^, which was shown to protect XPC from proteasomal degradation.

Recently, an additional ubiquitin E3-ligase; RNF111, that promotes XPC ubiquitylation was identified^21^. RNF111, also known as Arkadia, was originally named after the Arkadia mutation in mice. Homozygous *Arkadia* mutants are non-viable since they fail to form the regulatory primitive node, which is crucial during early gastrulation. This problem in the development of the mouse embryo is most likely caused by the loss of the ubiquitin ligase activity of RNF111 that promotes transforming growth factor-β signalling^22^,^23^. RNF111 belongs to the class of SUMO-targeted ubiquitin ligases (STUbLs), which facilitate crosstalk between SUMOylation and ubiquitylation. Accordingly, RNF111 specifically targets SUMOylated XPC and modifies it with K63-linked ubiquitin chains dependent on the E2 conjugating enzyme UBC13 (ref. 21). Altogether, these observations illustrate the importance of ubiquitin and ubiquitin-like modifications in regulating the DNA damage recognition factors that initiate NER^24^. In this study, we investigated the molecular function of the RNF111-dependent ubiquitylation of XPC and its role in NER. We show that although RNF111 is not essential for GG-NER, it strongly enhances the repair reaction by stimulating the release of XPC from damaged DNA, thereby enabling the progress of the NER reaction by recruitment of the endonucleases XPG and XPF/ERCC1.

## Results

### RNF111 is required for efficient GG-NER

**Figure 1:**
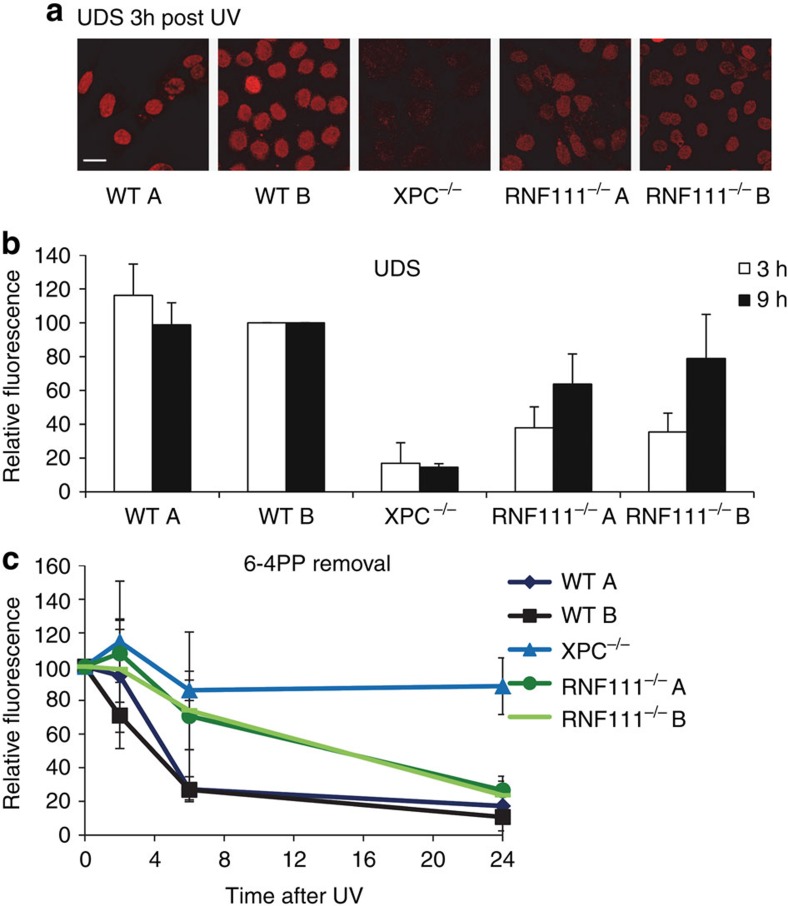
RNF111 is necessary for efficient GG-NER. (**a**) Representative pictures of unscheduled DNA synthesis (UDS) of the indicated MEFs, determined by 5-ethynyl-2′-deoxyuridine (EdU) incorporation over 3 h after UV irradiation (16 J m^−2^). Scale bar, 25 μm. (**b**) Quantification of UDS levels in MEFs, as determined by EdU incorporation over a time period of 3 or 9 h after UV irradiation (16 J m^−2^). UDS levels in WT MEFs were set at 100% (*n*>100 cells per sample, in at least two independent experiment; error bars are the mean±s.d.). (**c**) 6-4PP removal assayed by immunofluorescence, using a 6-4PP specific antibody. The indicated MEFs were UV-irradiated (10 J m^−2^) and allowed to repair 6-4PPs for the indicated time points. Relative fluorescence directly after UV exposure was set at 100%. (*n*>70 cells, three independent experiments; error bars are the mean±s.d.).

To study the role of RNF111 during NER we first determined the repair capacity in the absence of RNF111 by measuring the UV-induced unscheduled DNA synthesis (UDS) in the first 3 h after UV-induced damage^23^, which is a measure of GG-NER activity. In line with a previous study^21^, NER-deficient Xpc^−/−^ MEFs and Rnf111^−/−^MEFs (clone A and B) displayed a strongly reduced repair capacity as compared with NER-proficient wild-type (WT) MEFs (Fig. 1a,b). To test whether this reduced repair capacity in Rnf111^−/−^ MEFs is caused by a blocked or delayed NER reaction, 6-4PP repair kinetics were determined in WT, Xpc^−/−^ and Rnf111^−/−^MEFs. Cells were fixed at different time points after UV irradiation (10 J m^−2^) and immunostained for 6-4PPs. In WT MEFs the vast majority (≈75%) of 6-4PPs was removed within 6 h after UV irradiation (Fig. 1c, Supplementary Fig. 1a). As expected, 6-4PP repair was not observed in GG-NER-deficient Xpc^−/−^ MEFs, not even after 24 h. Rnf111^−/−^ MEFs displayed an intermediate phenotype; 6 h after UV irradiation 6-4PP removal was severely inhibited, with ∼70% of these lesions remaining (Fig. 1c, Supplementary Fig. 1a). Strikingly, 24 h after UV exposure 6-4PP repair was almost completed, suggesting that the NER reaction is not fully blocked, but rather seems to be retarded. This was further corroborated by measuring UDS levels over an increased time window of 9 h instead of 3 h. UDS levels of Xpc^−/−^ MEFs remained low, indicative of their full repair deficiency. However, residual UDS levels in Rnf111^−/−^MEFs increased from 40% over 3 h, up to 60–80% after 9 h as compared with WT MEFs (Fig. 1b, Supplementary Fig. 1b). Altogether, these results indicate that although RNF111 is not essential for GG-NER, but it strongly enhances the repair reaction.

### RNF111 is required for XPC release from sites of UV damage

**Figure 2:**
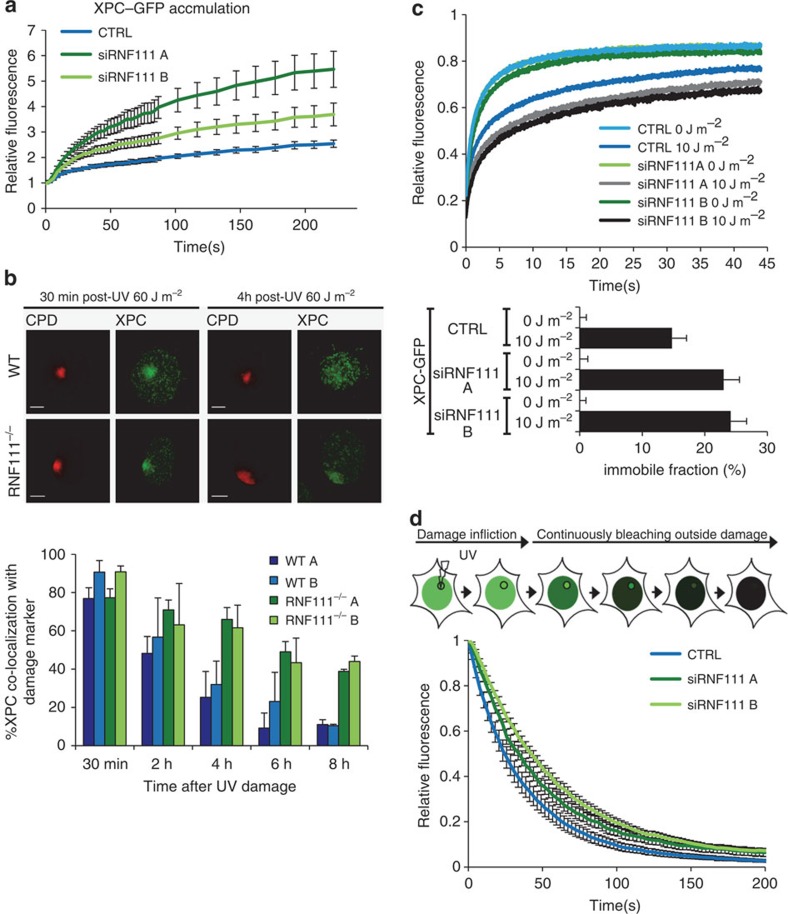
RNF111 is required for XPC release. (**a**) Relative XPC–GFP accumulation at sites of LUD in control and RNF111-depleted cells. GFP fluorescence intensity at UV-C laser induced LUD was measured over time using live-cell confocal imaging and quantified to predamage intensity set at 1 at *t*=0 (*n*>15 cells per sample, measured in two independent experiments; error bars are the mean±2 × s.e.m.). (**b**) Top panel: representative immunofluorescence pictures of co-localization of XPC with CPD at LUD in WT and Rnf111^−/−^MEFs at the indicated time points after UV irradiation (60 J m^−2^) are shown. Scale bars, 5 μm. Lower panel: quantification of the XPC co-localization with CPD (*n*>50 cells with LUD were analysed per sample in three independent experiments; error bars are the mean±s.d.). (**c**) Top panel: FRAP analysis of XPC–GFP in mock treated or global UV-irradiated (10 J m^−2^) XP4PA (XPC deficient) cells, on transfection with the indicated siRNA’s. XPC–GFP was bleached in a small strip within the nucleus and fluorescence recovery was measured over 45 s and normalized to prebleach intensity (*n*=40; from two independent experiments error bars are the mean±2 × s.e.m.). The immobilized fraction (%)=1−((average fluorescence intensity UV-irradiated cells−the first data point after bleaching)/(average fluorescence intensity unchallenged cells−the first data point after bleaching)), is plotted in the lower panel. The immobilized fraction was calculated over the last 10 s. (**d**) Inverse FRAP (iFRAP) analysis of XPC–GFP at LUD. XP4PA cells stably expressing XPC–GFP were transfected with the indicated siRNA’s. Seventy-two hours after transfection, cells were locally exposed to a 266-nm UV-C laser. After the accumulation plateau was reached (5 min after exposure) the undamaged part of the nucleus was continuously bleached and fluorescence in the damaged area was monitored. Fluorescence was normalized to prebleach intensity (*n*>15 cells per sample, measured in two independent experiments; error bars are the mean±s.e.m.).

RNF111 ubiquitylates XPC in response to UV exposure^21^. Therefore, the reduced GG-NER capacity in the absence of RNF111 suggests that RNF111-dependent ubiquitylation facilitates GG-NER by regulating XPC function. To further investigate this, we measured XPC–GFP accumulation kinetics at sites of local UV-C laser (266 nm) induced DNA damage (LUD) using quantitative live-cell confocal imaging. Surprisingly, knockdown of RNF111, using two independent siRNAs (Supplementary Fig. 1c) resulted in a twofold increase in XPC–GFP accumulation at LUD (Fig. 2a, Supplementary Fig. 1d). These data argue for an increase in XPC binding to lesions in the absence of RNF111 and suggest an improved DNA damage detection, which is seemingly at odds with the observed reduction in repair capacity in the absence of RNF111. The RNF111-dependent XPC binding properties were further investigated by determining long-term binding of XPC to DNA damage by scoring XPC co-localization with a damage marker (anti-CPD) using immunofluorescence (Fig. 2b). At 30 min after local UV irradiation, no difference in co-localization of XPC with LUD was observed. However, at later time points (4, 6 and 8 h) after UV a strikingly higher co-localization with LUD was observed in Rnf111^−/−^ MEFs as compared with WT MEFs (Fig. 2b). Similar results were observed in U2OS cells treated with two different siRNAs targeting RNF111 (Supplementary Fig. 2). In contrast, knockdown of RNF4, another STUbL involved in the mammalian DNA damage response^25^,^26^, had no effect on the UV-induced co-localization of XPC with DNA damage (Supplementary Fig. 2), showing the specificity of RNF111 for XPC regulation. This increased XPC accumulation could either be explained by a more stable binding of XPC to DNA damage or by a higher concentration of substrates, as RNF111 loss resulted in slower repair kinetics, or both. To distinguish between these possibilities and to resolve the apparent contradiction between more XPC binding and slower repair, we determined XPC mobility using fluorescence recovery after photobleaching (FRAP) on XPC–GFP in RNF111-depleted cells. The mobility of XPC–GFP was unaffected by RNF111 depletion under unperturbed conditions (Fig. 2c, 0 J m^−2^), indicating that the probing of DNA by XPC in the absence of UV-lesions is not affected^27^. On UV exposure (10 J m^−2^) XPC is engaged in damage recognition, resulting in an increased XPC–GFP immobilization^27^. Knockdown of RNF111 resulted in a further increase in XPC–GFP immobilization after UV, as shown by the FRAP curves (Fig. 2c, upper panel) and by plotting of the calculated immobile fractions (Fig. 2c, lower panel). These data suggest that XPC is more strongly associated with damaged DNA in the absence of RNF111, which might be a consequence of increased association (*K*_on_) and/or decreased dissociation kinetics (*K*_off_). To study whether the *K*_off_ is affected, we applied inverse FRAP to measure the dissociation of XPC from sites of DNA damage. To this end, LUD was first introduced to locally accumulate XPC–GFP until steady state was reached. Subsequently, the entire nucleus, with exception of the damaged area, was continuously bleached and the loss of fluorescence at the site of damage was measured at regular time intervals (Fig. 2d). Depletion of RNF111 resulted in reduced dissociation kinetics (*K*_off_) of XPC–GFP at LUD (*t*_1/2_=33–43 s) as compared with control transfected cells (*t*_1/2_=24 s), indicative of an increased residence time. Altogether our results suggest that RNF111 plays an important role in promoting the release of XPC from damaged DNA. The reduced clearance of XPC from damaged sites may thus explain the increased accumulation of XPC–GFP at LUD and may cause the delayed repair.

### RNF111 is essential for efficient XPG and XPF/ERCC1 loading

**Figure 3:**
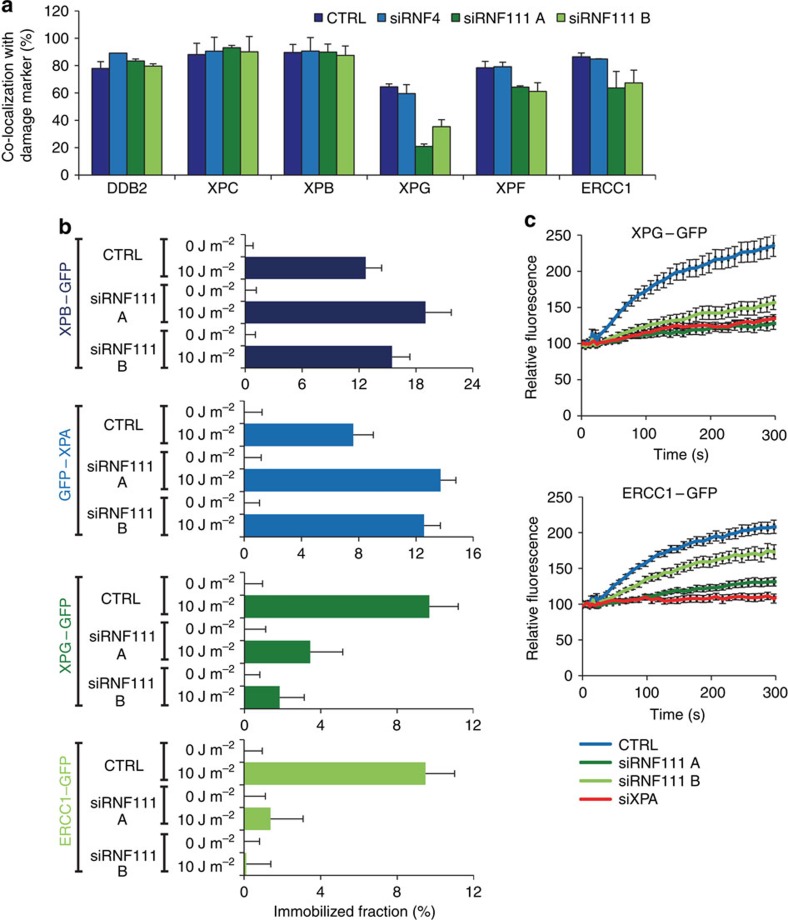
RNF111 is required for binding of XPG and XPF/ERCC1 to the NER complex. (**a**) U2OS cells expressing CPD–photolyase–mCherry were transfected with the indicated siRNA’s 3 days before the immunofluorescence experiment. Cells were local UV irradiated (60 J m^−2^) and immunostained for the indicated proteins 30 min later. The percentage of co-localization with the damage marker CPD–photolyase–mCherry at LUD is plotted in the graph (*n*>50 cells containing a LUD were scored in at least three independent experiments; error bars are the mean±s.d.). (**b**) The immobilized fraction of XPB–GFP, GFP–XPA, XPG–GFP and ERCC1–GFP as determined by FRAP analysis in mock or UV-treated (10 J m^−2^) cells on transfection with the indicated siRNA’s (*n*>32 cells from at least two independent experiments; error bars are the mean±2 × s.e.m.). (**c**) Cells stably expressing XPG–GFP and ERCC1–GFP transfected with the indicated siRNA’s were locally irradiated using a 266-nm UV-C laser. GFP fluorescence intensity at LUD was monitored for 6 min, with 10 s intervals and normalized to predamage values. (*n*=24 cells from three independent experiments; error bars are the mean±s.e.m.).

Our finding that knockdown of RNF111 results in prolonged binding of XPC to DNA damage provides a good model system to study NER factor handover during the repair reaction and to determine which NER factors depend on XPC release to be incorporated into the NER complex. To this end, we tested whether a panel of NER factors (DDB2, XPB, XPG, XPF and ERCC1) co-localized to LUD 30 min after UV irradiation (60 J m^−2^), as marked by CPD-photolyase-mCherry^28^, in RNF111 siRNA-depleted U2OS cells. Co-localization of early factors, like DDB2 (upstream of XPC) and XPB (subunit of TFIIH, directly downstream of XPC) with the DNA damage marker, was not affected by RNF111 knockdown. Interestingly, co-localization of the endonucleases XPG and XPF/ERCC1 with DNA damage was significantly lower (20–50%) in RNF111-depleted cells than in control siRNA-transfected cells (Fig. 3a). In contrast, depletion of RNF4 had no effect on UV-induced co-localization of NER factors with DNA damage (Fig. 3a). To study the *in vivo* binding characteristics of these factors to active NER complexes in the absence of RNF111 in a quantitative manner, we determined the mobility of these proteins by FRAP analysis in RNF111-depleted cells expressing GFP-tagged versions of XPB, XPA, XPG and ERCC1 after UV irradiation. Previous studies have shown that for each of these NER factors a clear UV-induced immobilization could be measured by FRAP^7^,^29^,^30^,^31^. Under unperturbed conditions (0 J m^−2^), no difference in mobility was observed for the indicated NER factors between control and RNF111-depleted cells (Fig. 3b). In contrast, after UV irradiation (10 J m^−2^) both the XPB and XPA proteins showed a further increased immobilization on RNF111 depletion, similar to what was observed for XPC (Fig. 2c). These results suggest that, like XPC, also XPB and XPA are more associated with DNA damage in the absence of RNF111. This increased association most likely represents longer dwell times of these factors into transiently trapped NER reaction intermediates that cannot finalize the repair reaction due to loss of RNF111. FRAP analysis of XPG–GFP and ERCC1–GFP in cells with reduced RNF111 levels revealed a striking opposite effect shown by a marked decrease in UV-induced immobilization, in line with the immunofluorescence experiments. This strong reduction of UV-induced immobilization likely reflects the inability of these endonucleases to stably integrate into active NER complexes in the absence of RNF111 (Fig. 3b). This was further confirmed by the strongly reduced UV-induced accumulation of XPG–GFP and ERCC1–GFP to LUD in living cells on RNF111 depletion, almost to the same extent as observed on siRNA mediated XPA depletion (Fig. 3c). As ERCC1/XPF binding to DNA damage is dependent on the presence of XPG^9^, these data suggest that when XPC remains bound to the initiating NER complex, both XPG and XPF/ERCC1 cannot be efficiently recruited to or stably incorporated in the NER complex.

### XPC release and ongoing NER is SUMO and K63-chain dependent

**Figure 4:**
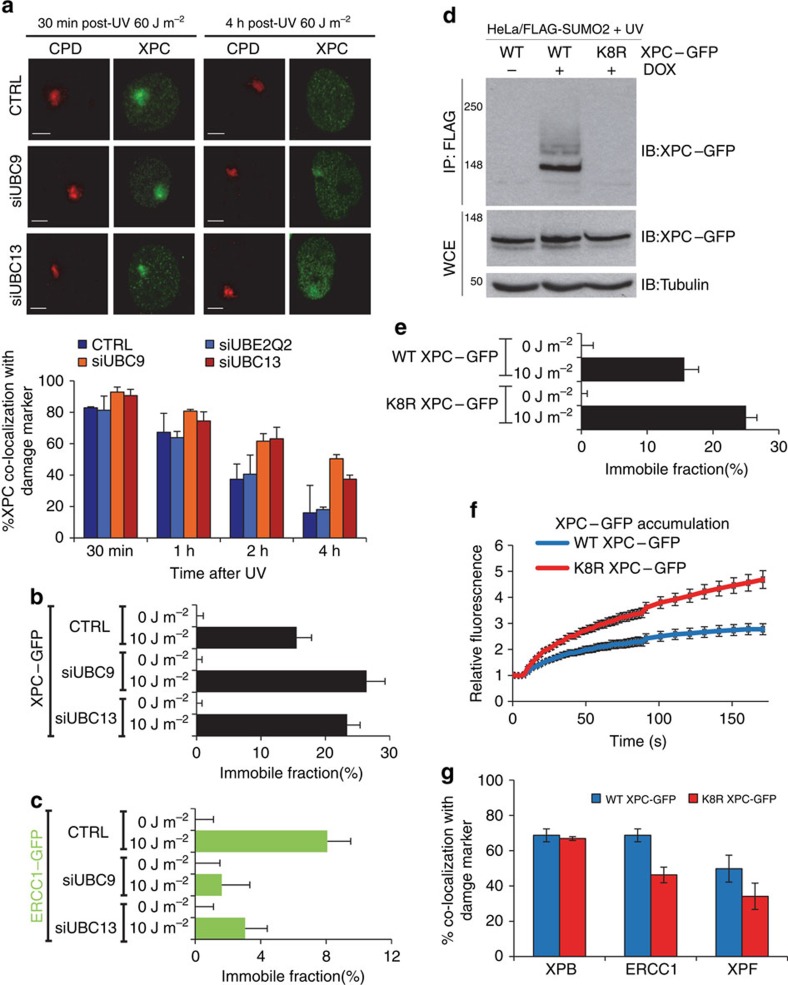
XPC release is SUMO and K63-ubiquitylation dependent. (**a**) Top panel: representative pictures of co-localization of XPC with CPD at LUD in U2OS cells transfected with the indicated siRNA’s 30 min or 4 h after local UV irradiation (60 J m^−2^) are shown. Scale bar, 5 μm. Bottom panel: quantification of XPC co-localization with the damage marker CPD. (*n*≈50 cells containing a LUD were scored per sample in three independent experiments; error bars are the mean±s.d.). The immobilized fraction of XPC–GFP (**b**) or ERCC1–GFP (**c**) as determined by FRAP analysis in mock or UV-treated (10 J m^−2^) cells depleted by siRNA of UBC9 or UBC13 (*n*=40 from two experiments; error bars are the mean±2 × s.e.m.). (**d**) HeLa/FLAG-SUMO2 cells were transfected with plasmids expressing WT or K8R XPC–GFP, then left untreated or incubated with doxycycline (DOX) to induce FLAG-SUMO2 expression. One hour after UV exposure (16 J m^−2^), cells were lysed under denaturing conditions, and XPC SUMOylation was analysed by immunoblotting of FLAG IPs with GFP antibody. (**e**) The immobilized fraction of WT XPC–GFP or K8R XPC–GFP as determined by FRAP analysis in mock or UV-treated (10 J m^−2^) cells (*n*>40 from three experiments; error bars are the mean±2 × s.e.m.). (**f**) Cells stably expressing WT XPC–GFP or K8R XPC–GFP were locally irradiated using a 266 nm UV-C laser. GFP fluorescence intensity at UV-C laser-induced LUD was measured over time using live-cell confocal imaging and quantified to predamage intensity set at 1 at *t*=0 (*n*>25 cells per sample, measured in two independent experiments; error bars are the mean±s.e.m.). (**g**) XPC deficient XP4PA cells stably expressing WT XPC–GFP of K8R XPC–GFP were locally UV-irradiated (60 J m^−2^) and immunostained for endogenous XPB, ERCC1 and XPF proteins 30 min later. The percentage of co-localization with GFP–XPC at LUD is plotted in the graph (*n*>100 cells containing a LUD were scored in at two independent experiments; error bars are the mean±s.d.).

As RNF111 is a STUbL that mediates UV-induced K63-linked ubiquitylation of XPC^21^, we investigated whether K63-linked ubiquitylation is required for XPC release from DNA damage and subsequent recruitment of the NER endonucleases. Towards this goal, siRNA targeting UBC13, the cognate E2-enzyme promoting K63-linked ubiquitylation^32^,^33^, was used and XPC co-localization at LUD with CPD as damage marker was scored in U2OS cells at several time points after UV irradiation. UBC13 depletion, as confirmed by western blot (Supplementary Fig. 3a), resulted in prolonged XPC co-localization with sites of DNA damage (Fig. 4a, red bars), similar to the observations in Rnf111^−/−^ cells. In contrast, depletion of another E2 conjugating enzyme, UBE2Q2, which is not involved in K63-mediated ubiquitylation^33^ had no effect on UV-induced co-localization of XPC with DNA damage (Fig. 4a, light blue bars). RNF111-mediated XPC ubiquitylation is dependent on XPC SUMOylation^21^. Therefore, we depleted UBC9, the E2 conjugating enzyme crucial for SUMOylation^34^, which indeed also resulted in more XPC co-localization with LUD at later time points (Fig. 4a, orange bars). Moreover, FRAP analysis of XPC–GFP showed that depletion of either UBC9 or UBC13 resulted in an increased UV-induced immobilization (Fig. 4b), to a similar extent as seen for RNF111 depletion. In addition, FRAP studies on ERCC1–GFP showed a decrease in UV-induced immobilization on depletion of UBC9 or UBC13 (Fig. 4c), indicating that XPC release from damaged DNA and the subsequent stable incorporation of the NER endonucleases into the repair complex is not only dependent on RNF111, but also on SUMOylation and K63-linked ubiquitylation. To address whether the SUMO-dependent ubiquitylation of XPC itself is sufficient to explain the observed effects on the release of XPC and recruitment of the downstream NER endonucleases, we set out to generate an XPC mutant that was refractory to SUMOylation^20^,^21^. With this approach, RNF111-mediated XPC ubiquitylation would be inhibited without affecting RNF111 activity towards other putative substrates. Using the GPS–SUMO algorithm^35^ we identified eight putative SUMOylation sites in XPC (Supplementary Fig. 3b). By mutating each of the eight lysine residues present in these SUMO consensus sites to arginines, we obtained an XPC mutant (K8R XPC–GFP) that could no longer be SUMOylated (Fig. 4d). The K8R XPC–GFP mutant was stably expressed in XPC cells and its mobility and DNA damage kinetics were analysed using live-cell imaging. No difference in mobility was detected under unperturbed conditions (0 J m^−2^) as determined by FRAP. However, on UV-induced DNA damage (10 J m^−2^) the K8R XPC–GFP was more immobilized than WT XPC–GFP, to a similar magnitude as was observed after depletion of RNF111 or UBC9 (Fig. 4e). In line with this, the K8R XPC mutant showed an approximately twofold increase in accumulation at LUD compared to WT XPC. Finally, similar to RNF111 depletion, we observed a clear reduction in ERCC1 and XPF accumulation at LUD in K8R XPC–GFP compared with WT XPC–GFP expressing cells (Fig. 4g), while no difference in the localization of XPB was found. Altogether these experiments demonstrate that the XPC release and subsequent binding of the NER endonucleases is dependent on the SUMOylation of XPC.

## Discussion

The recent identification of RNF111 as a STUbL involved in UV-induced ubiquitylation of XPC^21^ has added another level of complexity to the ubiquitin-dependent regulation of this DNA damage sensor, as previously also CRL4^DDB2^ was identified as an E3-ligase complex acting on XPC^17^. Interestingly, while both ubiquitin ligase activities are required for efficient GG-NER, they may have opposing effects on XPC. Whereas CRL4^DDB2^-induced ubiquitylation has been suggested to increase XPC DNA-binding affinity *in vitro*^17^, we provide evidence that RNF111 and its cognate E2—UBC13—are required for efficient release of XPC from UV-lesions, which permits the progress of the NER reaction. How can XPC ubiquitylation by two different E3 ligases have such a diverse functional outcome? One obvious explanation is that these E3 ligases modify XPC with different types of ubiquitin chains. While RNF111 in cooperation with UBC13 generates K63-linked ubiquitin chains on XPC, the exact type of ubiquitin chains formed by CRL4^DDB2^ is currently unknown. However, in line with the finding that CRL4^DDB2^ autoubiquitylates DDB2 resulting in its subsequent degradation^36^,^37^,^38^,^39^, most CRL4-type ubiquitin ligases promote proteasomal degradation of their substrates^40^,^41^, which suggests that CRL4^DDB2^ might form K48-linked ubiquitin chains on XPC. If indeed XPC is ubiquitylated by K48 chains in response to UV to increase its DNA-binding affinity, other factors may shield or protect it from proteolytic attack. One such candidate is the XPC complex partner RAD23B, which is known to protect XPC from proteasomal degradation, already in non-UV-challenged cells^42^. Other proteins involved in the stabilization of XPC might be the deubiquitylating enzymes OTUD4 and USP7, which were shown to deubiquitylate XPC upon UV-induced DNA damage^43^,^44^. It will be interesting to study whether these deubiquitylating enzymes are only involved in the protection of XPC from proteolytic degradation or if they are also important for ubiquitin chain editing on XPC. In this latter scenario, XPC would first be ubiquitylated by CRL4^DDB2^ after which the K48-linked ubiquitin chains might be trimmed down to permit K63‐linked ubiquitylation by RNF111 on the same lysines modified by CRL4^DDB2^, resulting in the subsequent release of XPC from sites of DNA damage.

**Figure 5:**
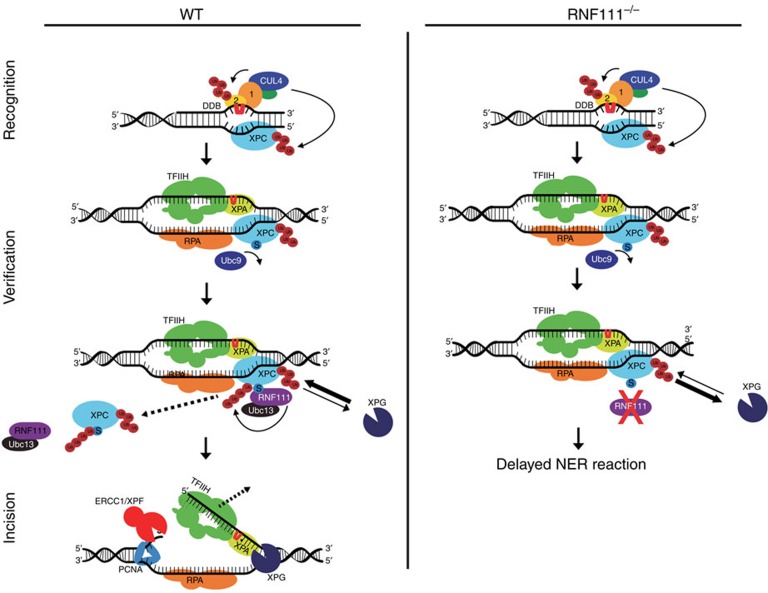
Proposed model for RNF111-dependent XPC ubiquitylation in controlling NER. In WT cells SUMOylated XPC is ubiquitylated by RNF111, promoting its release from damaged DNA. This RNF111-mediated XPC release facilitates XPG and ERCC1/XPF recruitment, thereby enabling an efficient NER reaction. In RNF111^−/−^ cells, where XPC is not modified by RNF111-dependent K63 ubiquitin chains, XPC remains more stably associated with the preincision NER complex. This interferes with proper loading of XPG, thereby inhibiting the NER reaction. Branched ‘Ub’ represents K48-linked Ub chains, linear ‘Ub’ represents K63-linked Ub chains; ‘S’ represents SUMOylation; ‘1’ and ‘2’ represent DDB1 and DDB2, respectively.

Intriguingly, the RNF111-mediated ubiquitylation occurs on one of the NER-initiating enzymes, but it affects one of the last NER steps; the loading of the endonucleases XPG and ERCC1/XPF. While we cannot exclude that RNF111 might also target other NER factors downstream of XPC that may contribute to the reduced NER-incision complex assembly, the reduced accumulation of ERCC1 and XPF at sites of UV damage in cells expressing an XPC mutant that cannot be SUMOylated (Fig. 4d–g) strongly suggests that this is caused by the action of RNF111 on XPC. In addition, no UV damage-induced SUMO modification of other NER factors have been described thus far. We therefore propose a model in which chromatin bound, SUMOylated XPC is ubiquitylated by RNF111 on DNA damage^21^, thereby stimulating its release from the NER preincision complex that contains TFIIH and XPA (Fig. 3b). This key step most likely generates better access of XPG or increased stable binding of XPG to the NER preincision complex. In addition, more efficient binding of XPG will promote the 5′ incision by ERCC1/XPF and progression of the NER reaction (Fig. 5, left panel). In contrast, in the absence of RNF111, damage-bound SUMOylated XPC is not ubiquitylated and remains stably bound to the NER complex, interfering with XPG loading and the subsequent recruitment of ERCC1/XPF, which are required for the excision of the damaged DNA strand by these endonucleases (Fig. 1b; Fig. 5, right panel). The need for XPC release for proper XPG incorporation into the NER complex is in line with *in vitro* experiments showing that on the arrival of RPA and XPG, XPC is released from the NER complex^45^. It should also be noted that the position where XPC is bound, the junction between double-stranded DNA and single-stranded DNA at the strand opposite of the lesion, 3′ with respect to the lesion-containing strand^10^, is also the site where the XPG endonuclease acts. For this reason of potential steric hindrance, it is logical to assume that XPC must be released before XPG loading.

Further research should uncover whether the ubiquitin-binding UBM domain present in XPG^46^ might play a role in this process. UBM domains have been shown to interact with monoubiquitin and K63-linked, but not K48-linked, chains^47^. This suggests that XPG might be able to interact with the K63-linked ubiquitylated form of XPC, generated by RNF111. In addition to the presence of TFIIH, this interaction could be required for efficient recruitment of XPG and for the subsequent or simultaneous extraction of XPC from the NER preincision complex^7^. Furthermore, it has been shown that XPG constitutively interacts with TFIIH^8^, which may suggest that TFIIH brings XPG into the NER complex. Interestingly however, and in line with earlier observations^7^, our data suggest that XPG is recruited to sites of DNA damage independent of TFIIH as it shows different RNF111-dependent binding kinetics than TFIIH: on RNF111 knockdown XPB is more stably immobilized on sites of DNA damage, whereas XPG immobilization could hardly be detected. However, another possibility is that XPG arrives as part of the TFIIH complex at sites of DNA damage, but will dissociate as long as XPC remains bound to the preincision NER complex.

The dynamic DNA association of XPC^27^ could give XPG the possibility to compete with XPC for binding to the NER preincision complexes even if XPC release is slowed down in the absence of RNF111-mediated ubiquitylation. This will eventually result in a functional NER reaction, however, at a much slower rate, which could explain the proficient, but strongly delayed, NER phenotype upon RNF111 knockdown (Fig. 1b,c).

On the basis of current knowledge it is expected that the different ubiquitylation events on XPC are regulated in a tightly coordinated manner to ensure that XPC binds and dissociates at the right time and place. Within NER, different—partially overlapping—stages can be recognized, for example, damage recognition and verification, establishment of the preincision complex and final dual incision. All steps before the actual incision are considered to be reversible, but once the incision by ERCC1/XPF is made the process reaches a ‘point of no return’^1^. We speculate that in response to UV, XPC is first modified by CRL4^DDB2^, resulting in more stable binding to sites of DNA damage. Subsequently, XPC is SUMOylated and recognized by RNF111, which mediates K63-linked ubiquitylation of XPC to promote its release from the NER complex. This XPC SUMOylation is dependent on the presence of DDB2 and XPA^20^. As XPA plays an important role during the damage verification step, it is expected that this XPC SUMOylation and its subsequent release occurs only after damage verification by the NER preincision complex (Fig. 5). We propose that RNF111-mediated ubiquitylation of XPC, required for stable integration of XPG, marks a decisive stage in the progression of NER reaction to reach the ‘point of no return’.

In summary, we have uncovered a new layer of ubiquitin regulation of the DNA damage recognition step of NER. We propose a first-in/first-out model: the ubiquitylation-driven release of the NER-initiating factor XPC is required to make room for the incorporation of the downstream NER endonucleases. This UBC13 and RNF111-dependent process is required to pass the NER reaction through the successive steps thereby facilitating efficient damage removal. In addition to the regulation by RNF111 and UBC13 as XPC ubiquitylation factors, this process is dependent on SUMOylation mediated by UBC9. This indicates the importance of crosstalk between SUMOylation and ubiquitylation in the regulation of damage recognition. Our findings not only show the importance of precise regulation of damage recognition, but also the regulation of the progress of the NER reaction. Taken together, we conclude that RNF111-mediated ubiquitylation of XPC is a key regulator of NER efficiency. The sequential SUMOylation and differential ubiquitylation of XPC to control the NER reaction might serve as a paradigm for the spatiotemporal regulation of other processes involving different types of sequential post-translational protein modifications.

## Methods

### Cell culture and treatments

U2OS cells were obtained from the ATTC cell collection, CPD–photolyase–mCherry^28^ was stably expressed by lentiviral transduction followed by Blasticidin selection. XPC–GFP was stably expressed in sv40 transformed XPC (XP4PA) cells by transfection of XPC–GFP^27^, XPB–GFP was stably transfected in sv40 transformed XPB (XPCS2BA) cells^29^, GFP–XPA was expressed in sv40 XPA (XP2OS) cells^30^ and XPG–GFP was stably transfected in sv40 transformed XPG (XPCS1RO)^7^ cells followed by FACS sorting and G418 selection. All cells above were cultured under standard conditions in DMEM/F10 supplemented with 10% FCS and 1% penicillin–streptomycin at 37 °C and 5% CO_2_. WT (clone A littermate of Xpc^−/−^and clone B littermate of Rnf111^−/−^), Xpc^−/−^ (ref. 48) and two independent clones of Rnf111^−/−^ knockout MEFs, indicated as A and B^23^ were grown in DMEM/F10 containing 10% FCS, 1% PS and 1% non-essential amino acids. HeLa cells stable expressing FLAG-SUMO2 in a doxycycline-inducible manner were generated by cotransfection of HeLa/FRT/TRex cells (Invitrogen) with pcDNA5/FRT/TO-3 × FLAG-SUMO2 and pOG44 followed by selection with 200 μg ml^−1^ Hygromycin B^21^,^49^.

For global and local UV irradiation cells were treated with a UV-C germicidal lamp (254 nm, Philips) at the indicated dose^50^. Local UV irradiation was applied through an isopore membrane filter (Millipore), containing 5-μm pores.

siRNA transfections were performed using hiperfect (Qiagen) or RNAiMax (Invitrogen)2–3 days before the described experiments according to manufacturer’s protocol. siRNA target sequence used were: CTRL (Thermo Scientific Dharmacon, D10-001210-05), RNF111(A) (5′- GGAUAUUAAUGCAGAGGAA -3′), RNF111(B) (Invitrogen, HSS182646), RNF4 (5′- GAAUGGACGUCUCAUCGUU -3′), UBC9 (5′- GGGAUUGGUUUGGCAAGAA -3′), UBC13 (5′- GAGCAUGGACUAGGCUAUA -3′), UBE2Q2 (Thermo Scientific Dharmacon, L-008326-01) and XPA (5′- CUGAUGAUAAACACAAGCUUAUU -3′).

### Construction and expression of ERCC1–GFP and K8R XPC–GFP

ERCC1–GFP was PCR amplified from pBluescript containing ERCC1–GFP-6xHIS-HA using the following primers: fw 5′- CCACATGGACCCTGGGAAGGACAAAG –3′ rv 5′- CTACTTGTACAGCTCGTCCATGCCGA —3′,cloned into pENTR-D-TOPO (Invitrogen) and recombined into the pLenti PGK Blast Destination vector (Addgene, plasmid 19065) using the Gateway LR Clonase II Enzyme Mix (Invitrogen). Third-generation lentivirus was produced in HEK293T cells and used to generate U2OS stably expressing ERCC1–GFP by Blasticidin selection. K8R XPC–GFP construct was generated by fusion PCR performed by Baseclear (Leiden, The Netherlands) and was sequence verified.

### Unscheduled DNA synthesis

Fluorescent-based UDS was performed as as follows: in short, MEFs were seeded on 24-mm coverslips 3 days before the UDS assay and cultured in serum-free medium to reduce the number of S-phase cells. Cells were UV irradiated with 16 J m^−2^ and incubated for 3 or 9 h in medium containing 5-ethynyl-2′-deoxyuridine (EdU; Invitrogen). Subsequently, cells were washed with PBS and fixed with 3.7% formaldehyde. Cells were permeabilized with 0.5% triton in PBS and 5-ethynyl-2′-deoxyuridine incorporation was visualized using Click-it Alexa Fluor 594 according to manufacturer’s instructions (Invitrogen). Images were obtained using a LSM700 microscope equipped with a 63 × oil Plan-apochromat 1.4 numerical aperture (NA) oil immersion lens (Carl Zeiss Micro imaging Inc.). Repair capacity, quantified in at least 100 cells by determining the overall nuclear fluorescence using ImageJ software, was normalized to fluorescence in WT cells, which was set at 100% (ref. 51).

### Live-cell confocal laser-scanning microscopy

For local UV-C irradiation in living cells, a 2 mW pulsed (7.8 kHz) diode pumped solid-state laser emitting at 266 nm (Rapp Opto Electronic, Hamburg GmbH) was connected to a Leica SP5 laser-scanning confocal microscope as described^52^,^53^. Cells were grown on quartz coverslips and imaged and irradiated at the indicated dose using an Ultrafluar quartz 100 × , 1.35 NA glycerol immersion lens (Carl Zeiss) at 37 °C and 5% CO_2_. Imaging medium was the same as culture medium. Images were acquired using the LAS AF software (Leica). Accumulation kinetics were quantified using FIJI image analysis software. Resulting curves were normalized to the relative fluorescence before irradiation and corrected for background values. To determine the dissociation kinetics of XPC from damaged DNA, the undamaged part of the nucleus was continuously bleached and the fluorescence decrease in the local damage was measured.

For FRAP analysis^54^, a narrow strip spanning the nucleus (512 × 16 pixels at zoom 8 was bleached for 100 ms using 100% of the power of a 488-nm laser. Recovery of fluorescence in the strip was monitored every 22 ms at 2% power of a 488-nm laser until fluorescence reached a steady-state level. All Frap data were acquired on a Leica SP5 laser-scanning confocal microscope equipped with a 63 × /1.4NA HCX PL APO CS oil immersion objective and normalized to the average prebleach fluorescence after subtraction of the background signal At least two independent experiments of >12 cells were performed for each condition. To determine the immobile fraction (*F*_imm_) from the FRAP measurements, we renormalized the data, using the fluorescence intensity recorded immediately after bleaching (*I*_0_) and the average fluorescence between 35 and 45 s after the start of the FRAP experiment (once recovery is complete) from the unchallenged cells (*I*_final, unc_) and UV-irradiated cells (*I*_final, UV_) and using the formula: *F*_imm_=1—(*I*_final, UV_—*I*_0, UV_)/(*I*_final, unc_—*I*_0, UV_).

### Western blot

Cells were collected by scraping in 200 μl 2 × sample buffer and boiled at 98 °C for 3 min. Lysates were separated by SDS–PAGE and transferred to a PVDF membrane (0.45 μm). Membranes were blocked with 5% milk in PBS for 1 h at room temperature and incubated with primary antibodies against RNF111 (H00054778-M05, Abnova), UBC9 (sc-5231,Santa Cruz Biotechnology), UBC13 (ab38795, Abcam) and Tubulin (T5286, Sigma Aldrich). Membranes were washed five times for 5 min with PBS containing 0.05% Tween and incubated with secondary antibodies from LI-COR to visualize antibody complexes with the Odyssey CLx Infrared Imaging System (LI-COR Biosciences). Uncropped scan of the western blots depicted in Fig. 4d can be found in Supplementary Fig. 3c.

### Immunofluorescence

Cells were grown on 24-mm coverslips and fixed using 2% paraformaldehyde supplemented with triton X-100. For XPG stainings, cells were fixed with 2% paraformaldehyde. Subsequently cells were permeabilized with PBS containing 0.1% triton X-100 and washed with PBS containing 0.15% glycine and 0.5% BSA. To visualize CPD or 6-4PP, nuclear DNA was denatured by incubation with 0.07 M NaOH for 5 min at room temperature. Coverslips were washed with PBS containing 0.15% glycine and 0.5% BSA and incubated with primary antibodies for 1–2 h at room temperature. Cells were washed three times and two times for 10 min with 0.1% triton X-100 and once with PBS containing 0.15% glycine and 0.5% BSA. To visualize primary antibodies coverslips were incubated for 1 h with secondary antibodies labelled with ALEXA fluorochromes 488 or 555 (Invitrogen). Again cells were washed with 0.1% Triton X-100 and PBS^+^. Subsequently coverslips were embedded in Dapi Vectashield mounting medium (Vector Laboratories). Images were obtained using a LSM700 microscope equipped with a 63 × oil Plan-apochromat 1.4 NA oil immersion lens (Carl Zeiss Microimaging Inc.). The following primary antibodies were used: anti-CPD(1:1,000; TDM-2;MBL International), anti-DDB2 (1:400; MBS120183 MybioSource), anti-XPC (1:200; fraction 5), anti-TFIIH p89 (1:1,000; S19; Santa Cruz), anti-XPG (1:400; 8H7; Thermo Scientific), anti-XPF (1:100; 3F2, Santa Cruz) and anti-ERCC1 (1:200; D10; Santa Cruz).

### Quantification of 6-4PP removal by immunofluorescence

MEFs were cultured to 80% confluence on 24-mm coverslips and exposed to global UV irradiation (10 J m^−2^). Cells were fixed after various time points and immunostained with anti-6-4pp (1:1,000; 64M-2; Cosmo Bio), as described above. Images were obtained using a Zeiss LSM 510 META confocal microscope equipped with a 63 × oil Plan-apochromat 1.4 NA oil immersion lens. 6-4PP levels were quantified in at least 70 cells per sample by measuring the overall nuclear fluorescence using ImageJ software, which was set at 100% for 0 h after UV irradiation.

## Additional information

**How to cite this article:** van Cuijk, L. *et al*. SUMO and ubiquitin-dependent XPC exchange drives nucleotide excision repair. *Nat. Commun.* 6:7499 doi: 10.1038/ncomms8499 (2015).

## Supplementary Material

Supplementary Figures Supplementary Figures 1-3
