# Supplementary material for: SUMO and ubiquitin-dependent XPC exchange drives nucleotide excision repair
Source: Nat Commun. 2015 Jul 7;6:7499. doi: 10.1038/ncomms8499 (PMC4501428; doi:10.1038/ncomms8499)
Supplement: Supplementary Figures — 1-3 [file ncomms8499-s1.pdf]

Supplemental figure 1

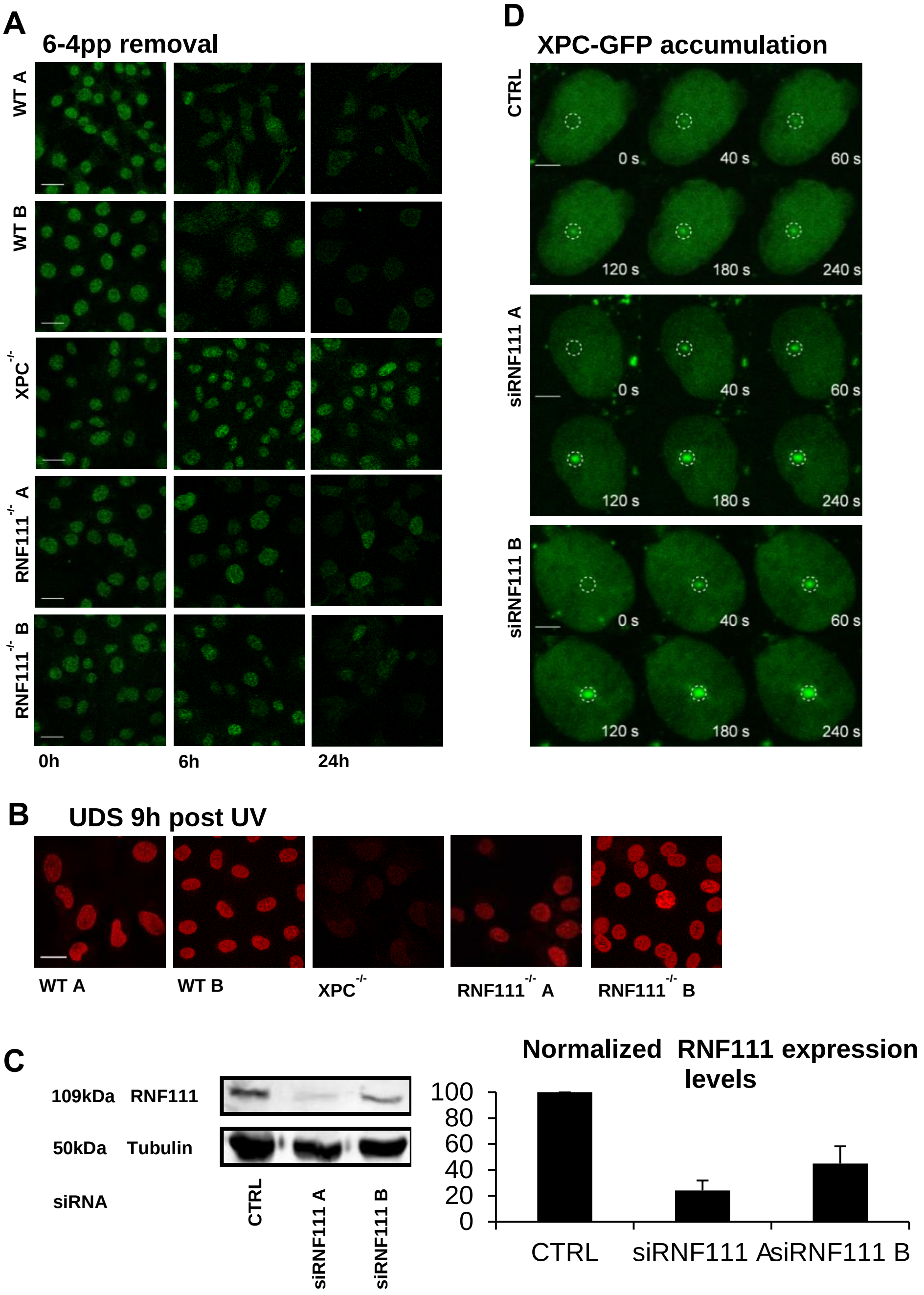

Supplementary Figure 1. (A) Representative pictures of the presence of 6-4PP in the indicated MEFs at the indicated times after UV-irradiation, measured by immunofluorescence. Scale bars: 25 µm. **(B)** Representative pictures of UDS of the indicated MEFs, determined by EdU incorporation over 9 h after UV-irradiation (16 J/m^2). Scale bar: 25 µm. **(C)** Left panel: RNF111 protein levels, as determined by western blotting, in U2OS cell were determined 72 h after siRNA transfection with non-targeting (CTRL) or RNF111 siRNA A or B. Anti-Tubulin staining was used as loading control. Right panel: quantification of the western blots of the left panel. **(D)** Representative pictures (stills) of live-cell imaging analysis of XPC-GFP after LUD infliction in XP4PA cells transfected with CTRL and RNF111 siRNA A or B. Dotted circle indicates the site of damage infliction.

Supplemental figure 2

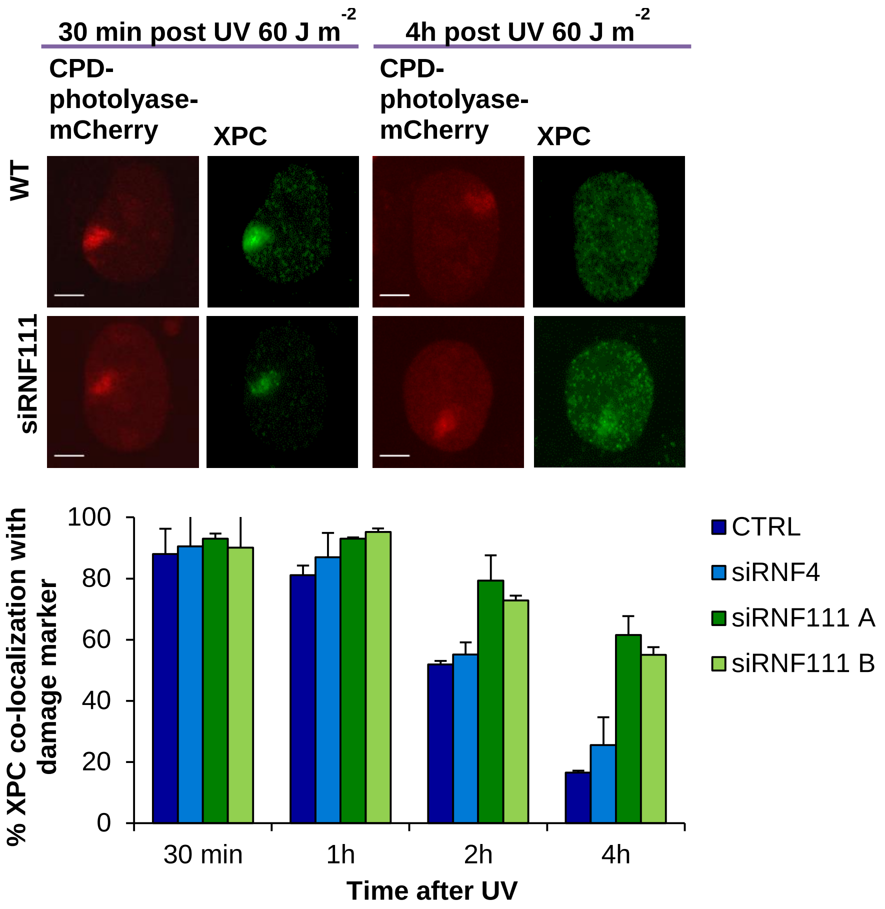

Supplementary Figure 2. Top panel: Representative pictures of localization of XPC and CPD-photolyase-mCherry at LUD in U2OS cells transfected with the indicated siRNA's 30 min or 4 h after local UV-irradiation (60 J/m^2) are shown. Scale bar: 5µm. Lower panel: Quantification of XPC co-localization with the damage marker CPD-photolyase-mCherry. (n≈50 cells containing a LUD were scored per sample in two independent experiments; mean ± SD).

Supplemental figure 3

A

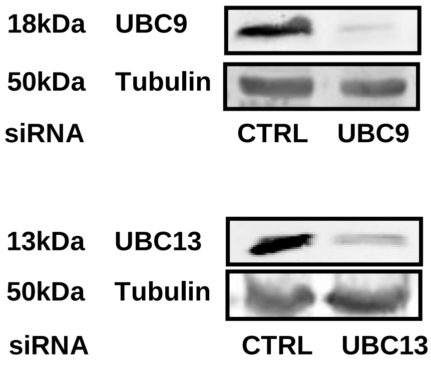

B

Position	Peptide	Score	Cutoff
4	MARKRAAGGEP	24.544	24.337
81	KVAKVTVKSENLKVI	48.354	15.22
89	SENLKVIKDEALSDG	45.653	15.22
183	RERSEKIKLEFETYL	41.732	15.22
564	TCYKYATKPMTYVVG	25.037	24.337
655	ALKRHLLKYEAIYPE	27.395	15.22
868	LKRRYGPKSEAAAPH	18.627	15.22
928	KKTKREKKAAASHLF	24.577	24.337

C

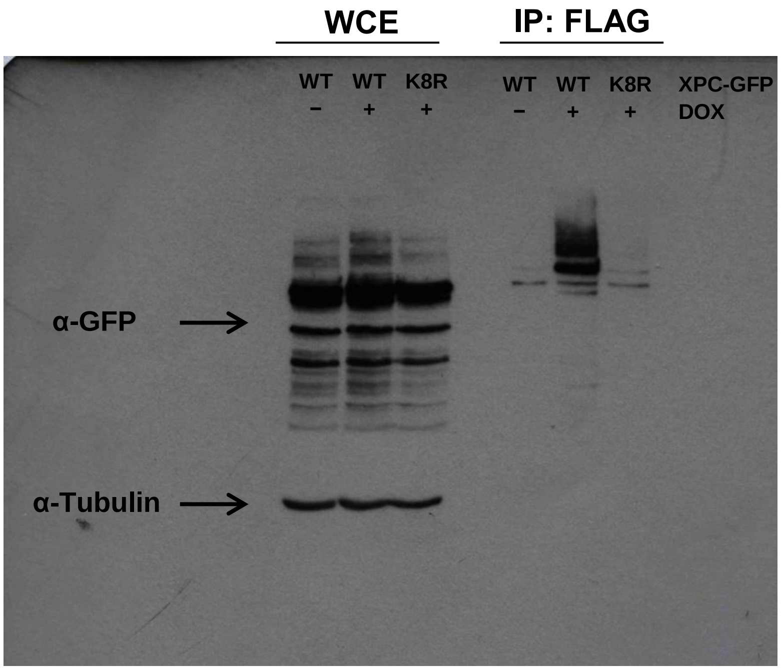

Supplementary Figure 3. (A) U2OS cells transfected with non-targeting (CTRL) siRNA or siRNA targeting UBC9 or UBC13 were collected 48 h after siRNA transfection. The amount of protein was analyzed by immunoblotting using the indicated antibodies. Anti-Tubulin was used as loading control. **(B)** Table showing the putative SUMOylation sites of XPC, as identified by the GPS-SUMO algorithm. **(C)** Uncropped scan of Figure 4D.
